# Effect of nursing interventions based on the Kano model on symptom relief and parental psychological behavior in children with febrile seizures

**DOI:** 10.3389/fpsyg.2022.1067727

**Published:** 2023-01-18

**Authors:** Zaiyun Zhu, Liping Chen, Kai Li

**Affiliations:** ^1^Department of Pediatrics, The Fourth Hospital of Changsha City, Changsha, China; ^2^Disinfection Supply Center, The Fourth Hospital of Changsha City, Changsha, China; ^3^Department of Rehabilitation Pain, The Third Hospital of Changsha City, Changsha, China

**Keywords:** febrile convulsions, Kano model, nursing intervention, symptom relief, psychological behavior

## Abstract

**Purpose:**

To analyze the effect of nursing interventions based on the Kano model on symptom relief and parental psychological behavior in children with febrile seizures (FS).

**Methods:**

A total of 104 children with FS and their corresponding families admitted to our hospital from January 2021 to April 2022 as the research object. All children were divided into 2 groups according to their nursing regimen during treatment. Children who received general nursing interventions were enrolled in the general group (*n* = 52) and children who received nursing interventions based on the Kano model were enrolled in the Kano group (*n* = 52). In this study, an investigation was first conducted to analyze the attributes of the caring care service needs of the families of children with FS. Then, we compared 4 aspects of symptom relief during the hospital stay of the 2 groups of children, including FS seizure frequency, time to cessation of convulsions, time to recovery of consciousness and time to fever reduction. The parent symptom questionnaire (PSQ) was used to assess the psychological behavior of the two groups of children during the hospital stay. The Chinese perceived stress scale (CPSS) and the symptom checklist 90 (SCL-90) were used to assess the psychological behavior of the two groups of their families during the children’s hospitalization. Finally, a questionnaire was administered on the satisfaction of this nursing intervention.

**Results:**

In terms of symptom relief, the children in the Kano group had less frequent of FS seizure than the general group, and the time to cessation of convulsions, time to recovery of consciousness and time to fever reduction were all earlier than in the genera group (*p* < 0.05). In terms of children’s psychological behavior, the impulsivity-hyperactivity, anxiety, hyperactivity index and learning problems scores in the PSQ of the children in the Kano group were lower than those in the general group after the intervention (*p* < 0.05). In terms of family psychological behavior, the psychological behavioral problems of the families of the children in both groups improved after the intervention, and the CPSS scores of tension and dis-control, as well as the total SCL-90 score of the families of the children in the Kano group were lower than those of the general group (*p* < 0.05). In terms of family satisfaction, the Kano group was significantly better than the general group (*p* < 0.05).

**Conclusion:**

The implementation of the nursing interventions based on the Kano model for children with FS was successful in dramatically reducing the clinical signs and symptoms of the children and meeting the psychological and behavioral needs of the children and their families.

## Introduction

Febrile seizures (FS) are the most common convulsive disorder of infancy and childhood, usually occurring within 24 h of the onset of fever, and with obvious age-related and self-limiting (Christensen et al., 2021). The onset of FS is often unpredictable, with the child suddenly experiencing loss of consciousness, rolling of the eyes, clenching of the teeth, cyanosis of the lips, foaming at the mouth, rigidity of the limbs, general or partial limb convulsions, and incontinence. The etiology of FS is complex, and the incidence of FS is high. Besides, the onset of FS usually occur suddenly when the family members of children are unprepared. The family members of children are more stressed, often accompanied by negative emotions such as irritability and anxiety (Yilmaz et al., 2008; Lee et al., 2016; Mosili et al., 2020; Wubetu et al., 2020; Bing et al., 2022), resulting in higher expectations for quality of nursing and the need for caring care, and increased difficulty for the nursing staff. Studies (Lee et al., 2016) have pointed out that taking targeted nursing intervention after fully understanding the nursing needs of children’s families can effectively correct the bad psychological behaviors of children’s families, which is extremely important for promoting children’s recovery and building a harmonious doctor-patient and nurse–patient relationship (Sakai et al., 2009).

With the comprehensive implementation of the family centered nursing concept, nurses should not only focus on nursing patients, but also pay attention to the needs of patients’ families, and explore the role and positive role of families in coping with patients’ diseases (Ji et al., 2021). The Kano model is a “two-dimensional cognitive theory” model of quality characteristic satisfaction based on the “two-factor theory” proposed by Japanese scholar Noriaki Kano (Materla et al., 2019), and is a research tool for classifying attributes of quality or demand. Kano model has changed the single dimension cognition of quality management in the traditional research field, and can investigate the service needs and demand attributes of research objects. In recent years, some foreign scholars (Hejaili et al., 2009; Zhang et al., 2013; Howsawi et al., 2020; Barrios-Ipenza et al., 2021) have applied it to research on improving patient satisfaction and improving hospital service quality, but such research in domestic medical field still needs to be developed. Based on the Kano model, this study investigated the nursing needs of the families of children with FS, mapped out the key areas for improvement in the nursing services provided by healthcare professionals, and further analyzed the impact of nursing interventions based on the Kano model on symptom relief and parental psychological behavior in children with FS.

## Materials and methods

### Research object

A total of 104 children with FS and their corresponding families admitted to our hospital from January 2021 to April 2022 as the research object. Inclusion criteria: The diagnostic criteria for FS were referred to the 2017 JSCN (Japanese Society of Child Neurology) guidelines (Natsume et al., 2017). Children diagnosed with FS by clinical examination. Children with axillary temperature ≥ 38°C or anal temperature ≥ 38.5°C with varying degrees of limb convulsions and impaired consciousness. Children with sound clinical data. Children whose families had signed an informed consent. And hospital ethics committee had approved the study. Exclusion criteria: Children with other causes of convulsions. Children with other cranial diseases or intracranial organic lesions. Children with other central nervous system diseases such as cerebral palsy, mental retardation, microcephaly, etc. Children with severe organ comorbidities. Children with a history of premature birth or birth asphyxia. Children with whose families had a communication, mental, cognitive or consciousness impairment and were unable to cooperate in completing the relevant questionnaire. All children were divided into two groups according to their nursing regimen during treatment. The group was divided into the general group (*n* = 52) and the Kano group (*n* = 52) according to the nursing regimen of all children during treatment. A general characteristics questionnaire was designed to investigate the child’s age, gender, only child or not, first hospitalization or not, and information about the child’s families such as gender, relationship with children, area of residence, education level, experience in caring for pediatric patients, and payment terms for hospitalization (Table 1).

**Table 1:** Comparison of general characteristics of children and their families in the general group and Kano group.

Characteristics	General group (*n* = 52)	Kano group (*n* = 52)	**χ**^2^/t	*P*
Children (*n* = 104)				
Age (years old)	3.94 ± 1.10	3.82 ± 1.05	0.569	0.571
Entry temperature (°C)	39.35 ± 0.47	39.31 ± 0.64	0.363	0.717
Gender (cases)			0.154	0.695
Male	28	26		
Female	24	26		
Only child (cases)			0.915	0.339
Yes	45	48		
No	7	4		
First hospitalization (cases)			1.321	0.250
Yes	47	43		
No	5	9		
Positive family history for FS (cases)			0.664	0.415
Yes	17	21		
No	35	31		
FS classification			0.248	0.619
Simple FS	41	43		
Complex FS	11	9		
Families of children (n = 104)				
Gender (cases)			1.377	0.241
Male	16	11		
Female	36	41		
Relationship with children (cases)			1.770	0.183
Parents	45	49		
Other relatives	7	3		
Area of residence (cases)			0.922	0.337
Urban	39	43		
Rural	13	9		
Education level (cases)			1.493	0.222
Primary school and below	0	0		
Middle-high school	36	30		
College and above	16	22		
Experience in caring for pediatric patients (cases)			0.624	0.430
Yes	31	27		
No	21	25		
Payment terms for hospitalization (cases)			0.650	0.420
Medical insurance	34	30		
Self-funded	18	22		

### Nursing methods

General group received general nursing interventions: (1) Provide a clean, tidy and welcoming ward environment for children. (2) Prepare emergency medicine and equipment in the ward to ensure that the child’s head leans to one side when convulsions occur, so as to avoid suffocation caused by inhaling secretions. (3) Inform the families of the child’s basic condition and medication precautions in accordance with conventional medical advice. (4) Strengthen monitoring of the child’s vital signs, temperature and convulsions, and communicate with the attending physician in a timely manner if there were any abnormalities.

Kano group received a nursing intervention based on the Kano model: (1) Design of a questionnaire on the attributes of the caring care service needs of the families of FS children: A seminar was organized for some senior medical and nursing staff in the department, and in combination with the results of relevant expert consultations and literature analysis methods, and based on previous treatment and nursing experience, a questionnaire was formulated on the attributes of the caring care service needs of the families of FS children based on the Kano concept. The questionnaires comprised 25 questions each on two sets of positive questions (provided/available) and negative questions (not provided/not available), both consisting of four dimensions: nursing skills (4 entries), service attitude (6 entries), quality nursing care (7 entries) and communication (8 entries). For example: “Entry 1 - Effective sedation prior to examination” was asked in the positive and negative questionnaire as “How would you feel if the nurse was able to sedate you effectively before the examination” and “How would you feel if the nurse was not able to sedate you effectively before the examination.” For each entry, there were 5 options: “satisfied,” “deservedly so,” “indifferent,” “tolerable,” and “dissatisfied.” In this study, the Cronbach’s alpha coefficient for the positive questionnaire was 0.802 and the content validity was 0.794, the Cronbach’s alpha coefficient for the negative questionnaire was 0.810 and the content validity was 0.781. Classification of demand attributes: According to the Kano model construction method, the quality demand attributes of nursing services were classified into 5 categories: must-be quality (M), one-dimensional quality (O), attractive quality (A), indifferent quality (I), and reverse quality (R). The Kano attributes of each entry were aggregated, and the attribute with the highest frequency was taken as the Kano attribute for that entry, and the current nursing content that needs urgent focus for improvement was filtered out accordingly. The criteria and results of this Kano attribute classification are shown in Tables 2, 3. For example, if the positive answers to a question are “satisfied” and the negative answers are “dissatisfied,” then the Kano attribute of the subject for this entry is O. (2) Improve the nursing process based on the Kano model: ① Ensure that must-be quality was met: Strengthen the professional training of nursing staff in the department, promote the nurses’ learning of theoretical knowledge and urge the consolidation and promotion of nurses’ nursing skills through educational lectures, organization of exchange meetings and technical competitions. Communication with the child’s family should be patient and respectful enough, using more respectful language, careful observation and care, solving the problems and difficulties encountered by the child’s family in a timely manner, using more soothing and encouraging words when communicating with the child, and adding smiles and gestures to the communication to close the communication distance and gain initial trust. Proactively collect the concerns of the child’s family and set up a question-and-answer model for study accordingly, and refine the survey items of nurse–patient communication satisfaction in the survey of child’s discharge satisfaction, and monitor the nurses’ implementation. ② Improve the satisfaction of one-dimensional quality: coordinate with the relevant departments, increase bedside screening equipment as much as possible, and simplify the process of auxiliary examinations. The department should issue a unified procedure guideline sheet and improve the various operating standards, after the treatment and examination, the nursing staff should know the situation of the children in time, and inform the family members of the changes of the children’s condition in time. Education on health and treatment precautions and instruction on simple nursing skills to involve the family in the care of the child. ③ Focus on attractive quality: put up cartoon posters and add recreational facilities (such as slide and seesaw) to optimize the hospitalization environment. Information fetch areas could be set up on each floor of the ward and in the waiting lobby, so that the families of children can keep abreast of related nursing matters. Play some cartoons appropriately in the waiting area to reduce the boredom and anxiety of children and parents when waiting for treatment. The head nurse conducted an inspection at the end of each month and summarized the results and problems of the previous phase of implementation in a departmental plenary session, pointing out items to be adjusted in the next phase to promote quality cycle improvement.

**Table 2:** Kano model attribute classification criteria.

Positive question	Negative question				
	Satisfied	Deservedly so	Indifferent	Tolerable	Dissatisfied
Satisfied	Q	A	A	A	O
Deservedly so	R	I	I	I	M
Indifferent	R	I	I	I	M
Tolerable	R	I	I	I	M
Dissatisfied	R	R	R	R	Q

M, must-be quality; O, one-dimensional quality; A, attractive quality; I, indifferent quality; R, reverse quality; Q, questionable results.

**Table 3:** Survey results on the attributes of the caring care service needs of the families of FS children.

Dimensions	Entries	Kano attributes
Nursing skills	Effective sedation prior to examination	M
	Effective sedation during seizures	M
	Timely notice of changes in the child’s condition	M
	No nursing complications occur	M
Service attitude	Patience in explaining family issues	M
	Kindness in communication with children	M
	Proactive service with a smile	O
	Proactive guidance upon inspection completion	O
	Nurse with gentle words	O
	Patiently calming the child during the operation	A
Quality nursing	Bedside examination of comatose children whenever possible	O
	Able to pacify children and prevent them from rejecting EEG monitoring^(*)^ equipment	O
	Involve family members in the care of children as much as possible	O
	Setting up a convulsion recovery hotline	O
	Play cartoons	A
	Play health education content	A
	There are dedicated platforms for accessing relevant knowledge	A
Communication	Able to provide detailed answers to questions about convulsions	M
	Able to answer in detail the role of tests, treatments and medication	M
	Able to explain in detail the side effects of anticonvulsant therapy	M
	Ability to answer family questions in detail and persuasively	M
	Able to keep the family informed of changes in the child’s condition	M
	Precautions to be taken prior to examination	O
	Instruct families in proper life care	O
	Help families understand the child’s behavioral responses	O

*According to the Expert Consensus on Diagnosis, Treatment and Management of Febrile Seizures (2017 Practical Edition) (Neurology Group of Pediatrics Branch of Chinese Medical Association, 2017), EEG examination and follow-up are recommended for children with FS who are accompanied by focal seizures, abnormal nervous system development, first-degree relatives with a history of idiopathic epilepsy, complicated FS and frequent seizures.

### Research indicators

In this study, we compared 4 aspects of symptom relief during the hospital stay of the 2 groups of children, including FS seizure frequency (the number of FS attacks in hospital), time to cessation of convulsions (time to cessation of convulsions during the child’s stay in hospital at one FS episode, or in the case of multiple episodes, the mean value was taken and included in the statistical analysis), time to recovery of consciousness (time to recovery of consciousness during the child’s stay in hospital at one FS episode, or in the case of multiple episodes, the mean value was taken and included in the statistical analysis) and time to fever reduction (time to cessation of fever during the child’s stay in hospital at one FS episode, or in the case of multiple episodes, the mean value was taken and included in the statistical analysis). The parent symptom questionnaire (PSQ) was used to assess the psychological behavior of the two groups of children during the hospital stay, which consists of 48 entries on 6 factors (impulsivity-hyperactivity, anxiety, hyperactivity index, learning problems, behavioral problems, psychosomatic problems), with each entry applying a 4-point scale from 0 to 3 point. The Chinese perceived stress scale (CPSS) and the symptom checklist 90 (SCL-90) were used to assess the psychological behavior of the two groups of their families during the children’s hospitalization, CPSS consists of 14 self-assessment items on 2 topics, namely, sense of tension score (items 1, 2, 3, 8, 11, 12, 14) and sense of dis-control score (items 4, 5, 6, 7, 9, 10, 13), each of which is rated on a 7-point scale from 1 to 7 point, and the sum of the dis-control score and the tension score is used to calculate the total psychological stress score of the child’s families, with the higher the score, the more severe the psychological stress, SCL-90 consists of 9 factors (anxiety, hostility, depression, paranoia, somatization, interpersonal, obsessive–compulsive, psychotic, and phobia) with a total of 90 entries, each of which is rated on a 5-point scale from 0 to 4 point, and the score for a factor in the scale is equal to the total score for each entry of that factor divided by the number of entries, the higher the score, the more severe the psychosomatic symptoms. Finally, a questionnaire survey was administered on the satisfaction of this nursing intervention, which was self-administered by experts in the hospital and included assessments of four dimensions: nursing skills, service attitude, quality nursing care and communication, a 10-point scale of 1 to 10 point was applied to each dimension, with scores of 10 to 8 point, 7 to 6 point and 5 to 1 point being satisfaction, basic satisfaction and dissatisfaction respectively, with a questionnaire Cronbach’s alpha coefficient of 0.820 and content validity of 0.797.

### Statistical analysis

Data analysis was done with SPSS 22.0. The measurement data ( $\bar{x}$ ±s) were tested by independent samples t-test. Count data n (%) was tested by the χ^2^ test. Graphical plotting was performed by GraphPad Prism 8.0. *p* < 0.05 was regarded as a statistically significant difference.

## Result

### Comparison of general characteristics of children and their families in the general group and Kano group

The general characteristics of the children and their families in the general group and Kano group were gathered for statistical analysis, and no statistical difference was found between the two groups (*p* > 0.05), which was available for comparative analysis (Table 1).

### Attribute analysis of the caring care service needs of the families of children with febrile seizures

The attribute classification criteria of the Kano model are shown in Table 2. The survey results on the attributes of the caring care service needs of the families of FS children are shown in Table 3.

### Analysis of symptom relief indicators for children

**Figure 1:**
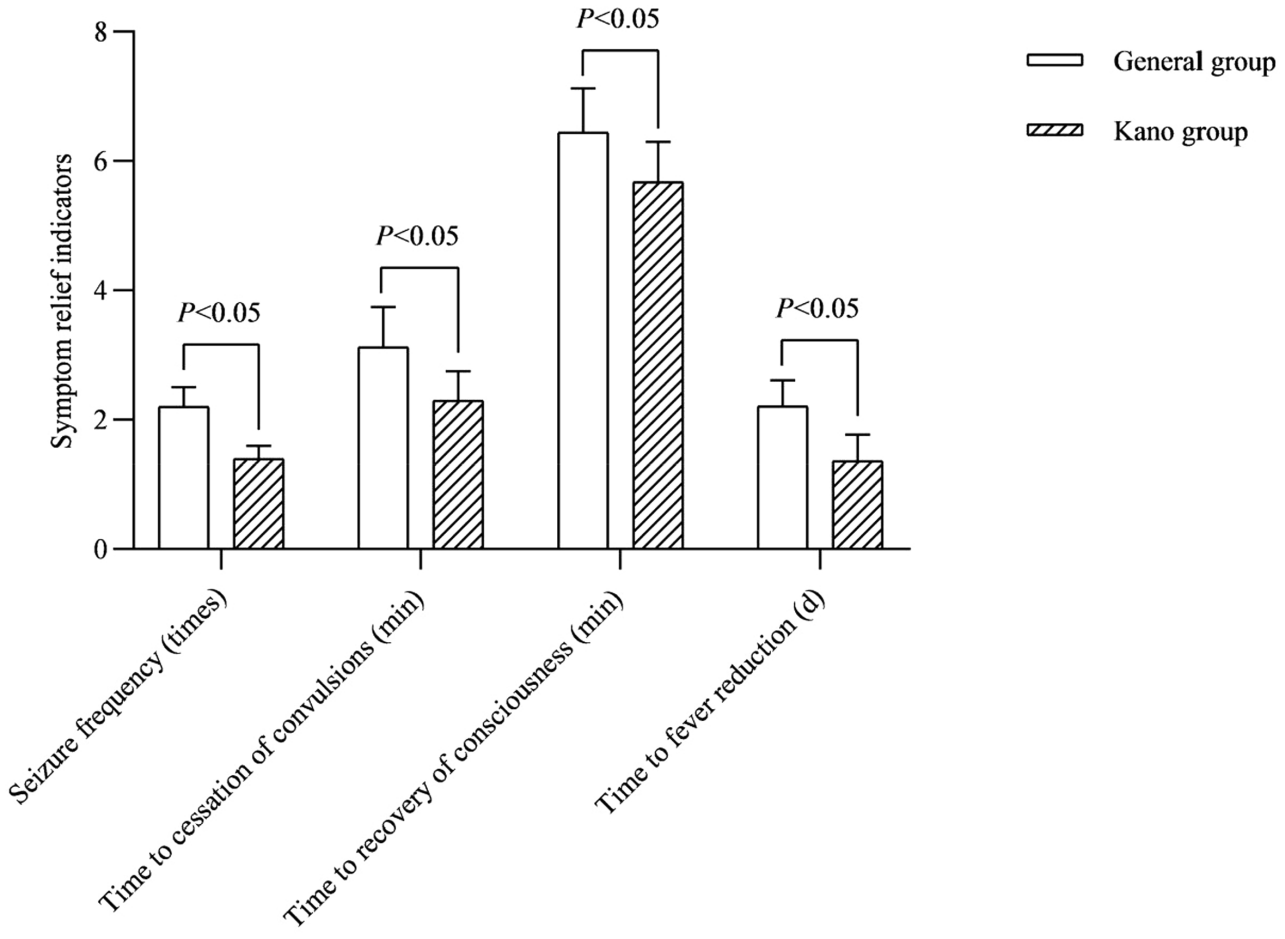
Symptom relief indicators for children.

In terms of symptom relief, the children in the Kano group had less frequent of FS seizure than the general group, and the time to cessation of convulsions, time to recovery of consciousness and time to fever reduction were all earlier than in the genera group (*p* < 0.05; Figure 1).

### Analysis of psycho-behavioral indicators for children

**Figure 2:**
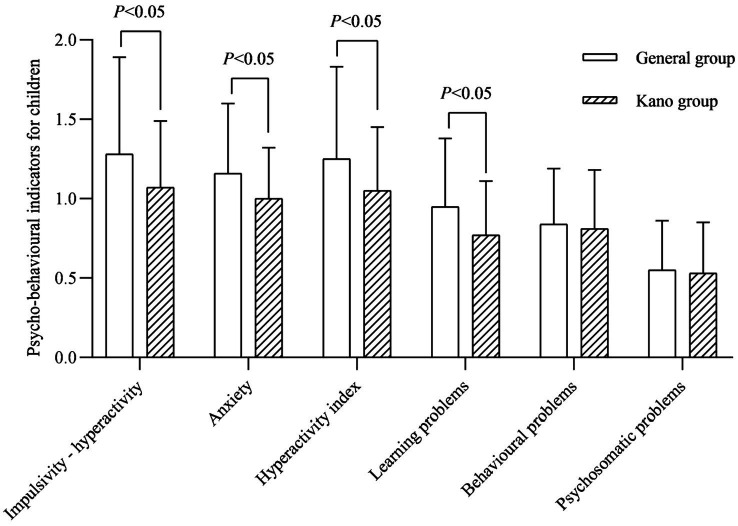
Psycho-behavioral indicators for children.

In terms of children’s psychological behavior, the impulsivity-hyperactivity, anxiety, hyperactivity index and learning problems scores in the PSQ of the children in the Kano group were lower than those in the general group after the intervention (*p* < 0.05; Figure 2).

### Analysis of psycho-behavioral indicators for families

**Figure 3:**
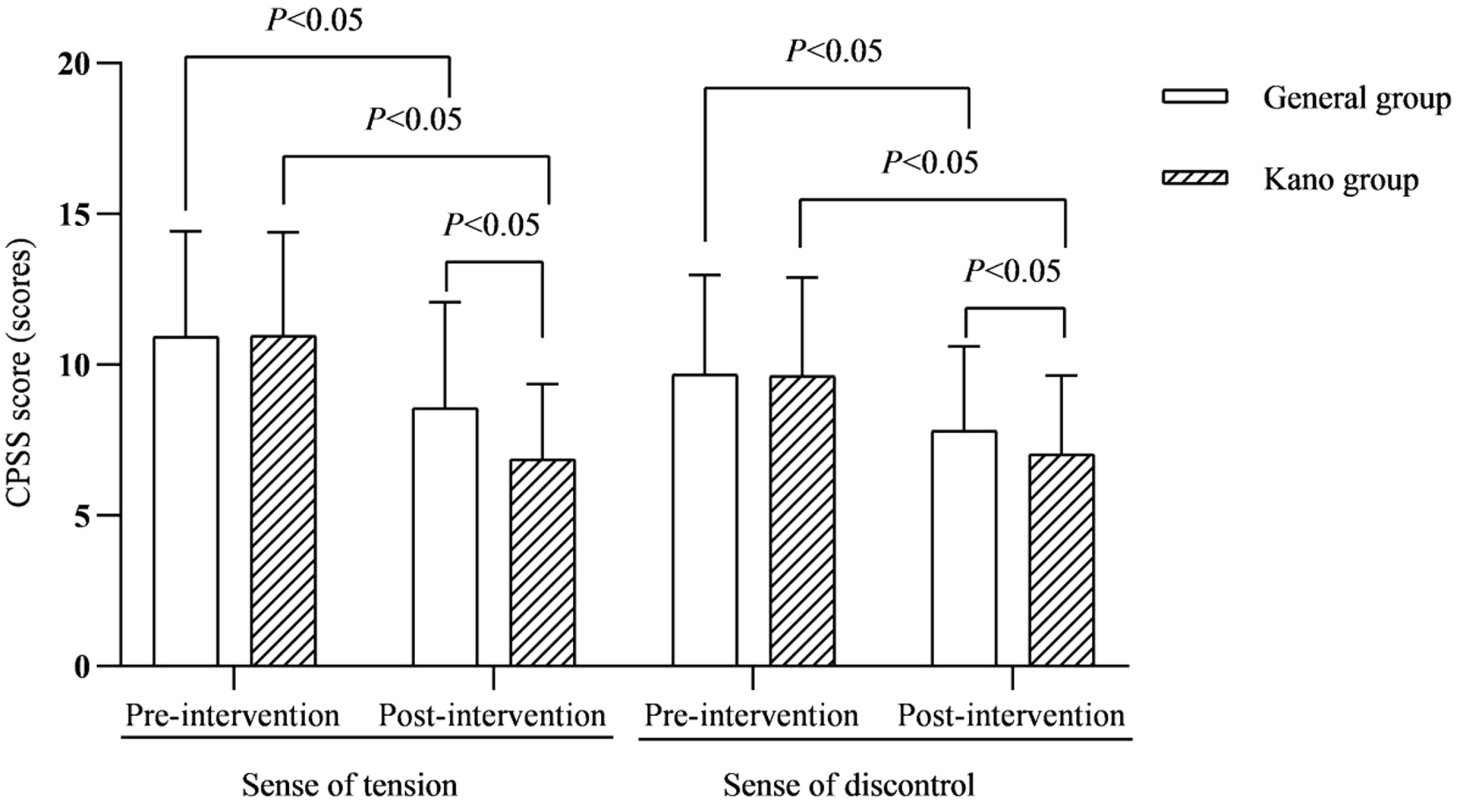
CPSS score (scores).

**Figure 4:**
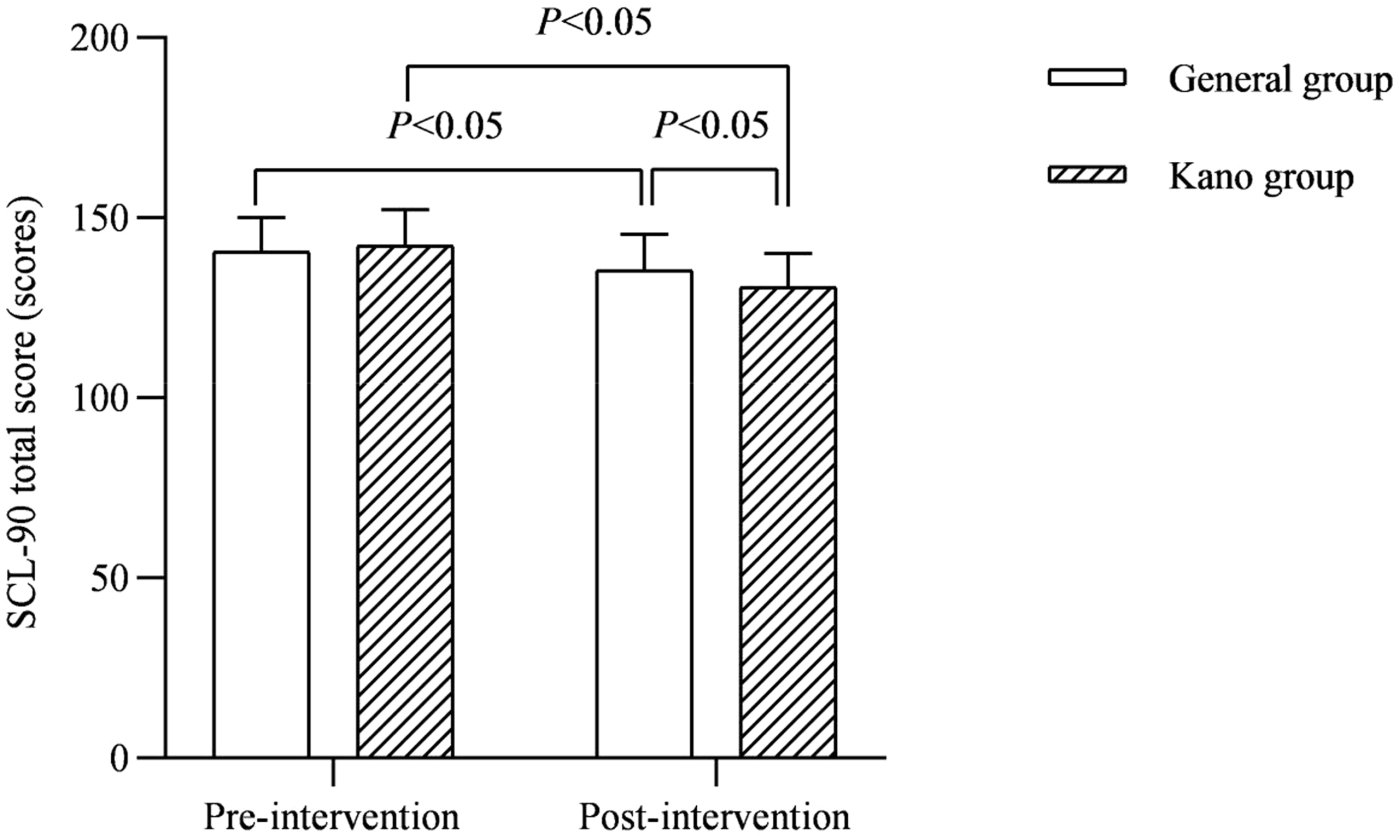
SCL-90 total score (scores).

In terms of family psychological behavior, the psychological behavioral problems of the families of the children in both groups improved after the intervention, and the CPSS scores of tension and dis-control, as well as the total SCL-90 score of the families of the children in the Kano group were lower than those of the general group (*p* < 0.05; Figures 3, 4).

### Analysis of satisfaction for families

**Figure 5:**
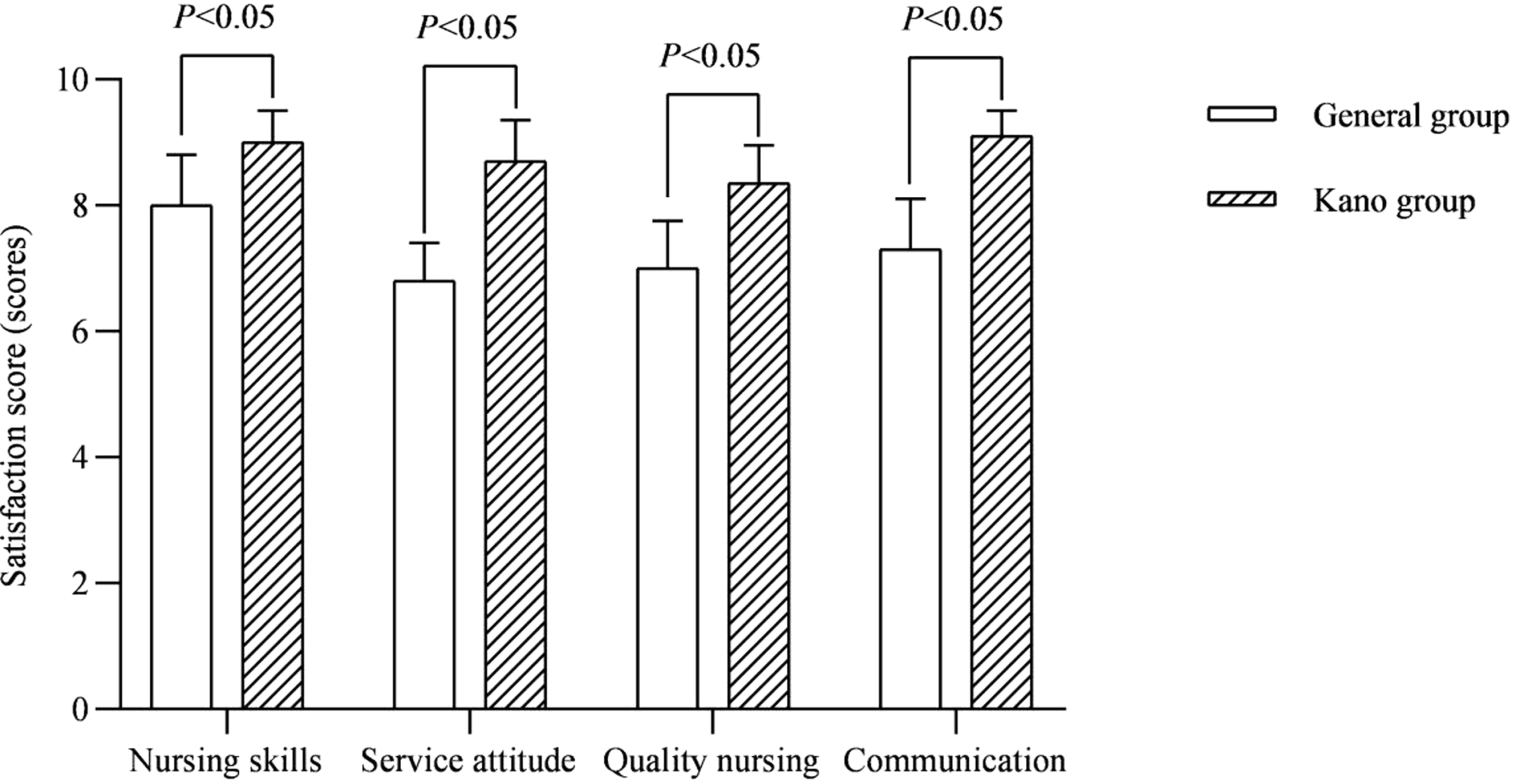
Satisfaction score (scores).

In terms of family satisfaction, the Kano group was dramatically better than the general group (*p* < 0.05; Figure 5).

## Discussion

The pathogenesis of FS is complex. Esmaili Gourabi et al. (2012) believed that it is closely related to genetic factors, 50.93% of FS children have a positive family history. In this study, the positive family history rates of the general group and Kano group were 32.69 and 40.38% respectively, slightly lower than the above study, which may be related to the difference of population characteristics in different regions. Among the various types of FS, simple FS (accounting for about 70–80% of all FS) is most often seen between the ages of 6 months and 5 years old, and rarely happens after 6 years old, with episodes usually lasting no more than 2 min, sometimes even just a few seconds, and rarely lasting up to 15 min, mainly presenting as full-scale seizures with no abnormal neurological signs, usually without damage to the brain, and with a good prognosis for the child (Mewasingh et al., 2020). Complex FS (accounting for about 20–30% of all FS) is most often seen under 6 months of age or over 5 years of age and is mainly focal, usually lasting more than 15 min and repeated several times, with neurological abnormalities in the child before and after the onset, some of which may turn into epilepsy, and therefore has a poor prognosis (Smith et al., 2019). Children with febrile status epilepticus (FSE) have recurrent episodes with states of impaired consciousness of up to 30 min or more between episodes (Jain and Santhosh, 2021). Prophylactic sedation during fever or regular long-term administration of anti-epileptic drugs can treat the children. Unfortunately, due to the lack of a comprehensive and systematic understanding of FS, most parents display a variety of abnormal psychological behaviors such as fear, dread, frustration and irritability at the early stage of the onset of the child’s illness, which has a certain degree of impact on the parents’ physical and mental health, and even on the outcome and prognosis of the child’s intervention (Bakula et al., 2021; Offringa et al., 2021). How to further improve the quality of pediatric nursing services has become a pressing issue in pediatric nursing management.

Based on the Kano model, this study divided the nursing service needs of family members into must-be quality (M), one-dimensional quality (O), attractive quality (A), indifferent quality (I) and reverse quality (R). According to the investigation results of family members of children, 3 dimensions of nursing that need to be improved urgently are screened out at present: must-be quality (11 items), one-dimensional quality (10 items), and attractive quality (4 items). After improving the corresponding nursing process based on the Kano model, the results showed that the children in the Kano group had significantly better symptom relief than the general group, and the children and their families had significantly better psychological and behavioral improvements than the general group. This may be because the nursing intervention based on Kano model can effectively avoid the blindness of nursing services, and medical staff can carry out targeted quality control accordingly, which can truly bring the nursing services required by patients to the best level.

Must-be quality refers to the need for care that the family members of children with FS believes is deserved. When this need is met, the family will not feel very satisfied because they believe it is deserved. On the contrary, if the need is not met, the family members will feel dissatisfied (González-Revaldería et al., 2017). The must-be quality items in this study primarily focus on the “nursing skills” and “communication” dimensions. This suggests that our nursing workers should not only pay attention to the learning of theoretical knowledge and the improvement of nursing skills, but also strengthen the communication with the family members of children to improve the quality of nursing and reduce the occurrence of medical disputes and complaints. In One-dimensional quality, there is a linear relationship between the degree of satisfaction of the child’s family and satisfaction, i.e., when that need is met, the child’s family feels satisfied and vice versa (Lin et al., 2022). The results of this survey show that the demand for “bedside examination of comatose children whenever possible” and “able to pacify children and prevent them from rejecting EEG monitoring equipment” is high. This suggests that our FS children’s families have high expectations for the convenience of various examinations for severe convulsions, especially for children in coma, therefore, in this dimension of nursing improvement, we need to focus on coordinating relevant examination departments, increasing bedside examination equipment as much as possible, and simplifying the auxiliary examination process. In addition, in view of the complex structure of EEG monitoring equipment for children with FS, there are many probes for children to wear, so the compliance is poor, how to appease children so that they do not reject real-time EEG monitoring equipment has not been a good solution at present, which is an important topic to continue to explore in the future nursing management research of FS children. Attractive quality is a potential demand that the children’s families themselves did not expect, if this demand is met, the overall satisfaction of the family members can be improved to a large extent, but if it is not met, the satisfaction will not be affected too much (Mao et al., 2021). In this study, the nursing measures that focus on attractive quality, such as optimizing the inpatient environment, setting up a information fetch area and playing cartoons in the waiting area, on one hand, meet the knowledge needs of the family and enable them to play a better monitoring role in the nursing process, on another hand, the improvement of the environment can also help to adjust the original tense atmosphere, improve the compliance of children, which in turn could fully exploit the effect of all treatment and nursing interventions, accelerating the relief of the child’s symptoms and contributing to the psychological behavior of the child and his or her family.

The Kano group was also significantly better than the general group in terms of family satisfaction. This may be due to the fact that the nursing service items of must-be quality, one-dimensional quality, and attractive quality for the child’s family are all met or addressed, the quality of care has improved significantly, thus increasing satisfaction. The Kano model differs from other models in that the implementer can analyze and target improvements to the original measures based on the subjective experiences of the service users, rather than the implementer or members of the research team analyzing the problems and making improvements on their own (Tripathi et al., 2017). Hence, the improved results based on the Kano model will certainly be highly relevant to the care needs of the children and their families. As a result, family satisfaction was greatly enhanced after the improvements were implemented.

## Conclusion

In a nutshell, the implementation of the nursing interventions based on the Kano model for children with FS was successful in dramatically reducing the clinical signs and symptoms of the children and meeting the psychological and behavioral needs of the children and their families. The limitations of this study are that the sample size is small, the survey is only conducted in one hospital, and a large number of suggestions from doctors and nurses are not consulted in the design of the questionnaire. Based on the results of this survey, the follow-up research will focus on continuously meeting the must-be quality, perfecting the one-dimensional quality, optimizing the attractive quality, and finally improving the service quality of family care for children with FS.

## Data availability statement

The original contributions presented in the study are included in the article/supplementary material, further inquiries can be directed to the corresponding author.

## Ethic statement

This study was approved by the ethics committee of our hospital. The patients/participants provided their written informed consent to participate in this study.

## Author contributions

ZZ was responsible for the writing and data analysis of the article. LC was responsible for research design. KL was responsible for ensuring that the descriptions are accurate and agreed by all other authors. All authors contributed to the article and approved the submitted version.

## Funding

This study was supported by Hunan Provincial Health and Health Commission Scientific Research Project (No. D202314046774).

## Conflict of interest

The authors declare that the research was conducted in the absence of any commercial or financial relationships that could be construed as a potential conflict of interest.

## Publisher’s note

All claims expressed in this article are solely those of the authors and do not necessarily represent those of their affiliated organizations, or those of the publisher, the editors and the reviewers. Any product that may be evaluated in this article, or claim that may be made by its manufacturer, is not guaranteed or endorsed by the publisher.
